# A Teleo-Reactive Node for Implementing Internet of Things Systems

**DOI:** 10.3390/s18041059

**Published:** 2018-04-01

**Authors:** Pedro Sánchez, Bárbara Álvarez, Elías Antolinos, Diego Fernández, Andrés Iborra

**Affiliations:** División de Sistemas e Ingeniería Electrónica (DSIE), Universidad Politécnica de Cartagena, Campus Muralla del Mar, s/n, 30202 Cartagena, Murcia, Spain; balvarez@upct.es (B.Á.); eliasantolinos@gmail.com (E.A.); diego.fernandez.alv@gmail.com (D.F.); andres.iborra@upct.es (A.I.)

**Keywords:** Internet of Things, Teleo-Reactive programming, multi-agent systems

## Abstract

The Internet of Things (IoT) is one of today’s main disruptive technologies and, although massive research has been carried out in recent years, there are still some open issues such as the consideration of software engineering methods and tools. We propose the adoption of the Teleo-Reactive approach in order to facilitate the development of Internet of Things systems as a set of communicating Teleo-Reactive nodes. The software behavior of the nodes is specified in terms of goals, perceptions and actions over the environment, achieving higher abstraction than using general-purpose programming languages and therefore, enhancing the involvement of non-technical users in the specification process. Throughout this paper, we describe the elements of a Teleo-Reactive node and a systematic procedure for translating Teleo-Reactive specifications into executable code for Internet of Things devices. The case study of a robotic agent is used in order to validate the whole approach.

## 1. Introduction

The Internet of Things (IoT) technology has many dimensions; it can be studied from multiple points of view and its growing popularity has come with an explosion in the number of protocols, tools, standards, architectures, middleware and platforms which have been developed to provide either vertical or horizontal solutions in many different domains [1,2]. The IoT is not just a promising technology, but also a growing paradigm that is the subject of massive research as it gains more and more adepts every day [3].

Despite the many advances made in recent years in solutions and platforms for the IoT, there are still many challenges whose solution remains unclear. One of them is the participation of the stakeholders in system specification, which might be solved by adopting the proper software modeling and implementation tools. Although IoT application development usually assumes that all the users participating in the process have analogous competencies and technological skills, this is not so, as role separation is a key concept in considering the multidiscipline perspective of IoT systems [4]. One relevant consideration is the heterogeneity of IoT devices, hampering the involvement of non-technical users and demanding sophisticated development methods to hide complexity and facilitate the understanding of system behavior [5]. Despite the contributions made by some researchers, the solutions are mostly focused on the use of general-purpose programming languages. The expected benefits from concern separation and the consideration of higher modeling abstraction levels are then lost. In this context, the adoption of the Teleo-Reactive (TR) approach is worthy to be explored for the specification of IoT systems. This paper presents the application of the TR approach as a way to facilitate the implementation of IoT systems.

The remainder of this paper is organized as follows: Section 2 briefly reviews the TR approach. Section 3 presents the state of the art of similar research. Section 4 describes the TR node, its structure of modules, and a systematic procedure to obtain Erlang code from the TR specification. In Section 5 the approach is validated by a case study. A discussion on the main results is given in Section 6. We conclude the paper in Section 7 by summing up the main points of the approach and outlining future research.

## 2. The Teleo-Reactive Approach

This section summarizes the basics of the TR approach. Nilsson at Stanford University introduced the TR paradigm in 1994 to systematize actions for autonomous agents in an environment susceptible to changes [6] and since then plenty of work has been done [7,8]. The TR approach allows agents to work in dynamic environments and to react to different environmental changes while they are aiming at a goal. These programs are conceived as a set of rules that lead a system to its final goal (hence *Teleo*) by constantly sensing the environment and responding to changes (hence *Reactive*) by triggering actions that ensure that the system always gets closer to its final goal. If desired, the final goal can also be modified when there are changes in the environmental circumstances. Table 1 depicts the basic structure of a TR program.

The *K_i_* are conditions on perceptual inputs and on a model of the environment, while the *a_i_* are actions on the environment or which change the environment. The list of rules is scanned from the top (where the rule with the highest priority is located) to the bottom (where the rule with the lowest priority is located). With the aim of being able to react to changes in the environment, the list of rules that compose the TR program is constantly evaluated. The first action executed belongs to the first rule whose condition is satisfied. The actions can be either single actions (durative or discrete) or another TR program, so that the hierarchical decomposition of goals is allowed. A discrete action is executed once the rule is activated, while durative actions are executed while the condition which has led to its execution is true and can be stopped and modified by the execution of other rules. 

**Figure 1:**
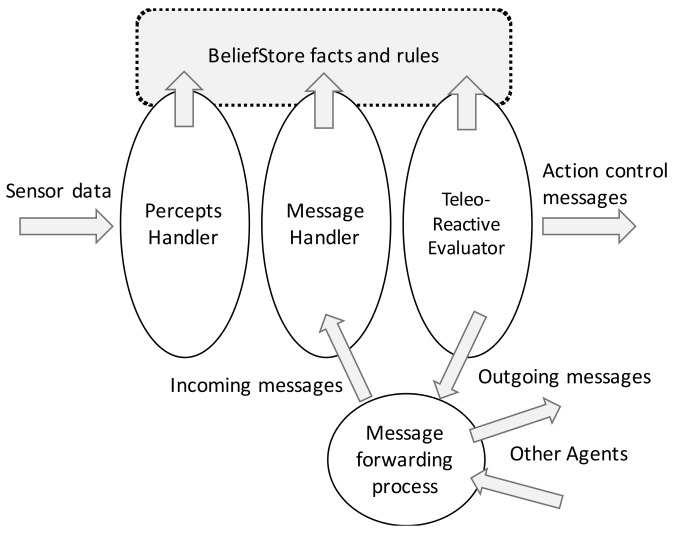
Multi-threaded TeleoR architecture (taken from [9]).

There is now an extension of the original TR language, TeleoR, which has support for execution [9]. TeleoR generalizes the architecture of the original TR proposal and presents a multi-task agent architecture in which four main elements can be clearly distinguished, as shown in Figure 1. The BeliefStore (BS) contains the agent’s knowledge, which is composed of facts and percepts deduced/taken from the environment. The Percept Handler thread converts sensor data into environmental information that is stored in the BS. Whenever a new message is received, the Message Handler thread stores a new belief (which is information inferred by the agent) in the repository. The TeleoR Evaluator then examines the BS to evaluate the guard of the rules in order to trigger the execution of the associated actions.

TeleoR extensions can optimize the semantics model and facilitate the specification of different actions. More specifically, TeleoR can manage variables of the agent used in the conditions and modify them as the result of the execution of a rule. As stated by Sánchez et al. in [10], some important extensions are: actions that include changes in the BS or send messages for concurrent executions with other agent actions, actions that are sequences of time limited durative actions (including procedure calls), actions that may be repeated if there are no changes in the environment in a specified time interval and resulting in the firing of another rule after a specified time, actions which allow the reception of messages from other agents and updating the BS, among others. For further information on TeleoR see the TeleoR website [11].

## 3. Related Work

This section focuses on existing studies in the literature that facilitate the lifecycle of IoT system development. One issue when developing IoT systems is how the behavior of the system is specified. On one hand, most well-known commercial solutions usually depend on general-purpose programming languages (i.e., Java, C, Python, etc.), which demand great effort and expertise from the developers and so hinder portability. In order to speed up the development of the IoT, the specification task should be simplified so that even end-users with a lack of formal software development training feel encouraged to act as auxiliary developers and able to specify and modify the behavior of their own systems. On the other hand, research proposals demand a great effort with a sharp learning curve because different modeling languages are considered, most of them as UML [12] diagram extensions.

One of the major contributions to supporting the whole lifecycle of software development is the Model Driven Engineering (MDE) approach [13], in which a core pillar is model manipulation, refinement and transformation, translating source models to executable target models. As the approach proposed here also considers the automated transformation from TR programs to code, it seems reasonable to compare this work with other approaches under the MDE umbrella in order to be able to put our contribution into perspective. The most representative contributions in this context are described below. For each of them a summary of the contribution of our work is given.

IoTSuite [5] adopts an approach based on macro-programming techniques and MDE, separating the application development into several concerns and integrating a set of high-level languages to specify them. Stakeholders can focus on the application logic by using the programming framework and use task-mapping and linking techniques to produce device-specific code. Despite being a sound framework for IoT development, its main drawback is that the implementation of the application logic is still done by general-purpose programming languages, implementing abstract methods of (automatically) generated abstract classes. DiaSuite [14] is a suite of tools providing support for designing, implementing, simulating and executing pervasive systems, closely related to IoT. It includes a language for architectural descriptions and entity discovery where component interactions are provided with high-level operations. Developers only use domain-specific concepts and notations, facilitating in doing so the design phase. However, the necessity of using the Java language for giving the application logic of the system and the considerable coding effort when heterogeneous devices are considered may incur an overhead for developers. PervML [15] allows users to develop pervasive systems using a set of UML-based models and a conceptual framework. The main drawback is that services are specified using UML sequence and state transition diagrams, which is again an overhead for developers and requires an additional effort to manipulate the diagrams. Other approaches that also rely on UML are [16,17], in which UML state transition diagrams are a common denominator to specify the application logic but an explosion in the number of states and transitions is usual and unmanageable. By way of example, all the transitions to deal with every possible alternative in the execution have to be explicitly considered. The worst part of these approaches is that the behavior of the system is given by means of general-purpose programming languages.

Despite the many initiatives to facilitate the development of IoT systems, we have not found any previous attempts at using the TR approach for IoT systems. TR programs provide a concise and precise way to specify the behavior of a system, as all the possible ways are implicitly modeled as part of the specification. Next section shows the structure of a TR node for the implementation of IoT systems, which minimizes the aforementioned limitations of current approaches.

## 4. The TR Node

We have conceived an autonomous IoT node whose software behavior is specified by a TR program and includes a mechanism for interpreting and executing its rules. The TR node is able to constantly sense and react to changes in the environment and, when many of these nodes work together, the TR architecture for IoT systems, which we have named TRIoT (TR for IoT), comes into play (see [18] for a preview of this architecture). Firstly, we will explain the architecture of the TR node and how its three functional modes work. By understanding this conceptual approach, developers should be able to customize their own TR node and adapt it to their particular application. For this, an example will be showcased of an implementation of the TR node using a TR interpreter, which translates from a basic subset of TeleoR to the Erlang programming language [19]. We have conceived a systematic procedure to achieve the translation and created a totally functional interpreter from scratch, whose validity was tested in a number of case studies. 

### 4.1. Hardware, Software and Functional Modes

**Figure 2:**
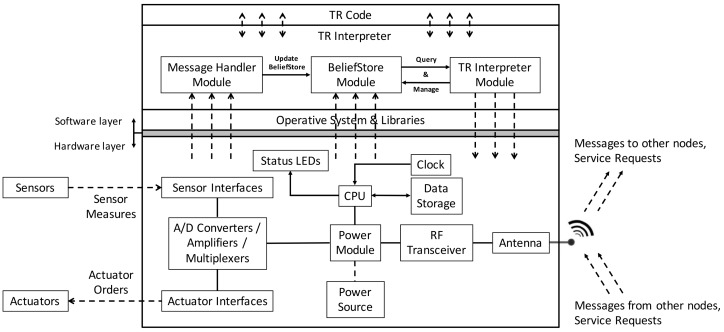
TR node modules.

Figure 2 shows details of the hardware and software of the TR node.

In terms of hardware, the node includes an antenna for RF reception and transmission which permits receiving and sending both messages and service requests, and a power supply regulation and management system that connects the device to a power source network or batteries. A power harvesting system (e.g., solar panel, wind generator, etc.) can also be attached. Depending on the node application, different sensors and actuators can be used (e.g., to monitor weather conditions and to open/close valves in a smart farming application) by means of a set of sensor and actuator interfaces. The TR node also has a module for conversion (analog to digital) and multiplexing the data read by the sensors or sent to the actuators, a clock that acts as a timer, status LEDs for notifications and alarms, a FLASH-type permanent read/write memory and a CPU/microprocessor to centralize the whole process and execute the TR behavior of the node. Since IoT devices are usually resource-constrained and do not have much memory, computation capacity or processing power, we considered a lightweight unit compatible with the usual IoT prototyping platforms for the TR node.

There are two main elements in the software layer: the TR code that defines the node behavior, which is given by the users, and the TR interpreter, which translates this TR code into executable code for the CPU. These two software levels are built over a third, which consists of the operative system and its libraries. The software levels can receive information through the hardware in the form of data measurements from the sensors and messages or service requests from other nodes through the antenna. The Message Handler module of the TR interpreter is in charge of updating the BeliefStore module according to the messages received. The software layer can also send messages to the hardware in the form of orders to the actuators and messages to other nodes or service requests through the antenna. This is mainly accomplished by the TR Interpreter module, which evaluates the rules of the TR program by querying the BeliefStore module to determine the rule of highest priority and trigger the execution of its associated actions. Since the TR node requires reproducing real world parallelism and concurrency, which involves several simultaneous actions, a concurrent programming language would be the best option to transfer the concepts of the TR paradigm. We implemented the TR approach using the Erlang programming language facilities (although other languages could also be considered). Erlang can build scalable soft real-time fault-tolerant, distributed, and non-stop systems with lightweight processes and therefore was the correct choice for our TR node. As the incremental cost per-process in Erlang is not high, it is possible to easily spawn hundreds of thousands of processes within a single application. Although there are Erlang Virtual Machines for some of the most popular devices used in the IoT (see http://www.erlang-embedded.com/), even though the Erlang Virtual Machine is lightweight, with the most resource-constrained embedded devices its needs could become an impediment.

The TR node can be parameterized and configured to work in three different functional modes, each with its own behavior and resources. Depending on its configuration, the TR node is named “Local Node”, “Local Coordination Node” or “Coordination Node”. When many nodes work together they will occupy different hierarchical levels inside the network architecture, according to their functional mode:

- Local Node (LN): When configured as an LN, the TR node assumes its simplest behavior and lowest hierarchical position in the TR network. In this situation, its main function is to sense the environment and to react accordingly. The node can also exchange messages with other nodes or ask for web services, although this is not its main purpose. It is a final node in the physical domain.
- Local Coordination Node (LCN): Configured as an LCN, the TR node adopts a middle hierarchical level in the TRIoT architecture. With this setup, the main purpose of the node is to coordinate the different LN networks and to act as a broker with external agents, services or other LCNs. Although it is not a must, sensing and actuating capabilities are also possible.
- Coordination Node (CN): This configuration places the node at the top of the hierarchy. When configured in this way, the node has no sensing or actuating capabilities and focuses its behavior solely on coordinating the different LCN networks, on serving as a broker with external agents, services or other CNs, and on connecting to the Internet. These nodes may also be virtually implemented and deployed instead of physically by using cloud-computing facilities.

**Figure 3:**
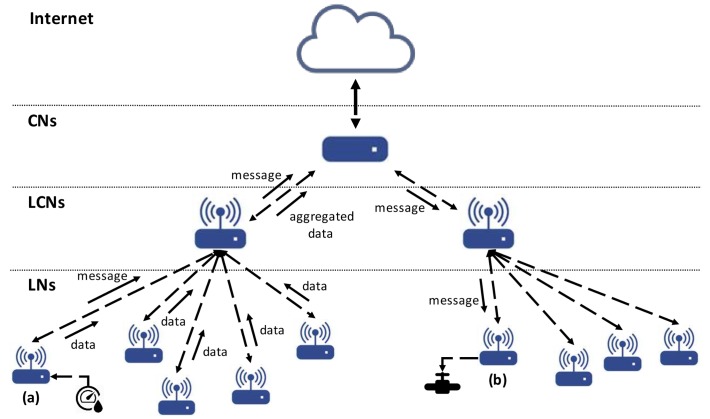
Example of a possible TR architecture based on the TR nodes. TR node (**a**) has a moisture sensor attached. Data measurements are assessed by the node and sent to the LCN, which gathers data from many different nodes and makes decisions or sends it to the CN. The CN can obtain data from both the LCNs and the Internet by service requests, and if the behavior is specified in its TR code, it can send an order to the TR node (**b**) to open or close the valve attached to it.

Figure 3 shows a diagram of a possible hierarchical architecture by using these functional modes.

The justification for this differentiation lies in the fact that as we go up in the hierarchy fewer sensors/actuators are involved but the usefulness of the TR approach is still valid. A developer could decide to consider a default implementation be implemented for the nodes in one level or node using TR programs but not in others. The type of messages used when implementing a CN node will be quite different to those for an LN node and perhaps also the type of attributes to be considered (such as those relating to security, robustness, privacy, etc.).

### 4.2. TR Interpreter

The TR Interpreter, which is able to translate TR code into the Erlang code run by the Erlang Virtual Machine, is together with the TR code the main software element of our TR node. The main goal was to design a procedure in which this translation could be performed systematically. As already stated, Erlang was chosen over other programming languages due to its advantages, especially in the way it handles concurrency. Concurrent programming can be used to improve system performance and make it more scalable and fault-tolerant. This TR interpreter implementation also uses the Open Telecom Platform (OTP), which is an Erlang framework composed of a set of modules and standards designed to help build the applications. Logan et al. [20] found that the main advantages of OTP are: productivity (systems can be created in a very short time), stability, supervision (the application structure makes it simple to control the systems), and reliability. 

**Figure 4:**
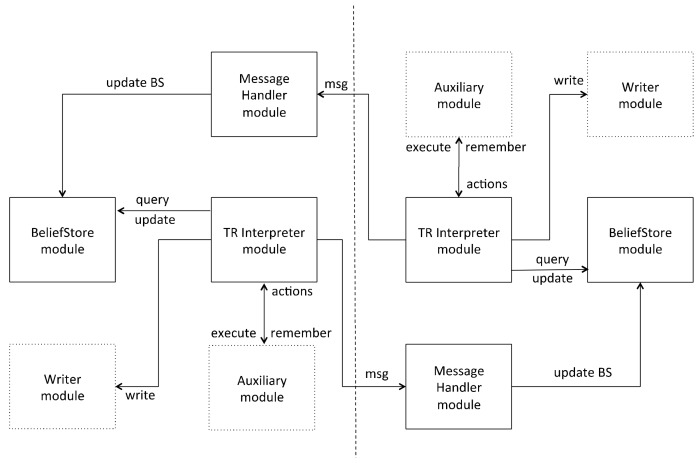
Communications between two TR nodes: modules involved in the implementation.

The TR interpreter consists of five different modules and it has been developed from scratch. Figure 4 shows an example of communications between two different IoT TR nodes, in which the TR interpreter has been implemented, and the three main modules involved: the Message Handler, BeliefStore and TR Interpreter modules. This module structure is compatible with those shown in Figure 1 and Figure 2, assuming that communication is by an LCN node.

The Message Handler module of one node receives messages from another node and, according to the information received, updates the node’s BS by adding, removing or modifying beliefs. The TR Interpreter module of each node evaluates the rules of the TR program by querying the BeliefStore module, decides the highest priority rule and triggers the execution of its associated action, e.g., to send messages to the other node. The Auxiliary module and the Writer module support the TR Interpreter module. The former contains a set of functions to control the execution of the TR program and simplify the implementation of the TR interpreter, while the latter is simply used to access the actuators of the device and to write in a log file (for debugging purposes).

The following subsections describe a case study of a TR node test and the functionalities of the Erlang interpreter, explain how these modules are implemented, and describe the algorithm developed to interpret the code. To clarify the operations of the interpreter and its translation algorithm, an example of the TeleoR code of a bottle collecting robot is given in Table 2.

This bottle collecting robot is similar to the one proposed in [21] by Dongol et al. and briefly reviewed in [7]. The robot’s job is to collect bottles and place them in a drop. In order to do this, it can rotate, move forward, scan the environment for bottles or for the depot and open or close its gripper. The robot has sensors to determine if it is seeing, touching or holding the bottle. For example, the robot will rotate looking for a bottle and once it finds it, and provided that the environment does not change, it will move forward until touching it, it will hold the bottle, turn until finding the depot and move forward to leave it there. Three parts can be distinguished in the TeleoR code: the declaration section, the goals and the message handler sections. The declaration section is where enums, percepts, beliefs, actions and variables are declared. In this example, the enums are the direction of the robot (left or right) and the things which the robot interacts with (bottle and drop). Percepts are pieces of information inferred by the agent (in this example they describe whether the robot has the grip open, if the robot is holding or touching the bottle, if the robot sees the bottle or if it is in position to leave the bottle). Non-percept beliefs that the agent wants to remember about what it has seen or done in the past, or has been told by another agent, or fixed facts about the environment and agent resources (not considered in the case study). This robot has five goals (collect_bottles, drop_and_leave, leave_drop, get_to_drop and get_bottle). Actions can be either discrete (open or close the gripper) or durative (move or turn the robot). Variables in this example are used to determine the number of bottles collected. The main goal collect_bottles is in charge of the behavior of the agent, accompanied by four subgoals. Every time a robot collects a new bottle, it sends a message to the other robot (addressable through the variable OtherAg), sending the number of bottles collected. Once the sum is reached (ten in the example) both robots finish the task. The address of each robot is given when calling the main goal _collect_bottles.

The following subsection describes the systematic procedure for translating the TeleoR code into Erlang code, once the invariable modules of the interpreter (i.e., the BeliefStore module and the Auxiliary module, explained in detail in Section 4.2.2 and Section 4.2.3, respectively) have been generated and the declaration section of the TeleoR program has been analyzed, in order to add variables to the BS and determine whether the functions are discrete or durative.

#### 4.2.1. TR Interpreter Module

The TR Interpreter module is in charge of controlling the execution of the TR rules by constantly evaluating all the rule conditions in order to select the activated rule with highest priority. It also controls executions of discrete and durative actions. Although Erlang facilitates much of the work (basically due to the structure of function definitions and the pattern matching facility) some issues do emerge regarding the termination and initiation of durative actions, control of timed operations, access to a centralized BS, determination of rule priorities, among others. We decided to present the compilation process by means of templates and small examples using the bottle collecting robot to give readers an easy way to see the transformation process. A deeper insight would need a good Erlang language background.

Four steps accomplish the interpretation as described below. Each step comes with an implementation example of the bottle collecting robot so that the algorithm is easier to follow. Erlang programs consist of functions that call each other and are grouped together and defined within modules. When a function is called, its clauses are checked sequentially by pattern matching the arguments passed in the call to the patterns defined in the function head. If the pattern match is successful, variables are bound and the body of the clause is executed. If not, the next clause is selected and matched.

1. First, the main structure of the TR program is created in Erlang by considering the goals. For each goal, the interpreter generates a new entry within the Erlang “case ... of” structure, as shown in Table 3. Thanks to the Erlang pattern matching mechanism, by which variables are bound to values by the evaluation of the conditions from top to bottom, the execution of the first satisfied condition is achieved.

The different conditions of the case expression are evaluated. As soon as a match is found, the corresponding action is evaluated. The result of evaluating the action is the value of the case expression. For each condition, there are two possibilities:

- If the condition is expressed on a percept or belief, then the Erlang condition checks if the percept/belief exists in the BS by using the function bs:is_belief, defined in the BeliefStore module. This function returns a true or false value when a belief exists or not, respectively (see Table 4).

Table 5 provides an example of how this is accomplished for the case of the bottle collecting robot.

The call for the discrete and durative actions occurs. Discrete actions are called by using auxilary:execute([Rule,Function,Args]), while durative actions are called by using auxilary:execute_while([Rule,Function,Args,Time]). Rule is an identifier composed of the name of the goal and a label to know which rule is initiating the action. This is needed because the same action (e.g., turn, move) could be started from different rules and the interpreter has to know which version is the action with highest priority. Durative actions are executed by indicating the function, its arguments and the duration of the execution (e.g., 2 s, as a rule can have a sequence of timed action). If the value of Time is set to ‘true’, the action is executed while the rule is activated. The main goal is called to repeat the evaluation of the algorithm. In this way, all the conditions are assessed again to determine which action should be triggered.

- If the condition compares a variable/belief/percept with a certain value or arithmetic expression, the implementation is made by using the function auxiliary:compare_value(Key, KeyList, Mode, Value), where Key is the belief/percept that is going to be compared and KeyList is the list where the belief/percept is stored. For variables, the function used is bs:get_belief, where bs is the instance of the BeliefStore module (see example in Table 6).

For the bottle collecting robot, this is implemented as shown in Table 7.

2. The second step is concerned with data management, and it consists of updating the variables/beliefs of the BS as a consequence of the execution of a rule. This process is accomplished by the function bs:update_belief (see Table 8).

Table 9 shows how this is done for the case of the bottle collecting robot. As it can be seen, the new value of the variable is passed as an argument to the call of the main goal.

3. The third step of the algorithm consists of remembering and forgetting beliefs as stated in the execution of the rules (for details see TeleoR [11]). These instructions are accomplished with the auxilary:remember(Belief,Time) function, the bs:remove_belief (which removes the whole belief set), and the bs:remove_one_belief function (which removes a value from a multi-value belief). For example, the robot could be able to see other robots and remember this fact with the ‘seen’ belief and for fifteen seconds.
4. The last step consists of managing the sending and reception of messages to/from other agents. The case study includes sending a message to the other robot with the number of bottles collected (count(_collected) to OtherAg). The message handler section in the TR program is in charge of processing the incoming messages, in this case storing the value in the _other_collected variable, which is used as part of the condition to stop the task. Table 10 shows the equivalence between both languages.

This easy node-to-node communication is direct given the facilities of Erlang for communication when one process knows the address of another. However, the question that arises now is how the communication between separate nodes could be managed in order to deploy the architecture showed in Figure 3. Our proposal relies on the existing IoT technology, which does all the underlying work. One feasible way to achieve that is by using EMQ (Erlang MQTT Broker: http://emqtt.io/), which provides a scalable, reliable, enterprise-grade PUB/SUB MQTT message Hub for IoT. EMQ is written in Erlang/OTP and licensed under the Apache Version 2.0. The EMQ broker is cross-platform, which could be deployed on Linux, FreeBSD, Mac, Windows and even Raspberry Pi. In this technology context, the Erlang implementation includes for each TR node a specific process in order to interact with the Message handler process. Each TR node connects through this process to an EMQ broker and subscribes to topics that it is interested in and according to the hierarchy of nodes. At the same time, the TR nodes connect to the broker and publish messages to topics, enabling the communication to all levels. 

#### 4.2.2. BeliefStore Module

The BeliefStore module is in charge of managing the state of the node and the environment. It has an OTP gen_server behavior (see [22]), since it is going to update the system’s state according to the changes in the agents and the environment, and is comprised of percepts and beliefs. Behaviors are a way of formalizing common patterns in process-oriented programming. The behavior interface is a specific set of functions and associated calling conventions. A behavior implementation is a callback module that exports the functions required by the interface. Erlang/OTP generically implements lots of networking paradigms, including finite state machines, event handling, and client/server interaction (the *gen_server* behavior). The client/server model is based on many clients connecting to a single, central server. The clients can send and receive messages from the server while the server maintains a global state.

The interfaces of the functions used to manage the BS are given in Table 11. Some of them are called in the implementation of the interpreter in Section 4.2.1.

Two different parts can be distinguished in the implementation of the BS: one belongs to the API definition (Table 11) and the other one belongs to the OTP gen_server definition. The API functions definition is shown in Table 12.

The start_link/0 function and the stop/0 function allow the server to control the BS to start and stop the gen_server, respectively. The other functions belong to one of these two groups: functions, which query the BS (get_belief/1, is_belief/1, is_belief/2 and get_bs/0) and functions which modify the BS (add_belief/1, update_belief/1, remove_one_belief/1 and remove_belief/1). The implementation of the gen_server functions is given in the GitHub repository (https://github.com/ppalma00/teleo-reactive-erlang) (to completely understand the implementation decisions, the reader will require basic knowledge of Erlang/OTP). The init/0 function initializes the BS state and includes some initial beliefs as specified in the TR program. In order to get the priority of each TeleoR condition, the belief priority has to have an ordered list of goals to determine the priority of each action. In each list of subgoals that belongs to the main goal there is an extra subgoal to compare main goals. The rest of the functions are separated into other two; handle_call and handle_cast, where only the input arguments change. Once the server has been started we want to be able to access the BS. We implement wrappers to handle these functions, each of which invokes gen_server:call/2. Thus, gen_server:call is used for synchronous communication between the client and the server, i.e., it is used when the server expects a response. These calls are handled by handle_call. It is possible to select the function to be executed by simply modifying the arguments thanks to the Erlang pattern-matching mechanism. In these cases, the function executed is determined by an atom (e.g., “stop”, “add”, “remove”, “remove_one” or “update”, for the handle_cast function).

Summing up, the greatest benefit of adopting gen_server for implementing access to the BS is the abstraction it provides, as it encapsulates the essence of the client/server model so that we can focus on the business logic rather than low-level event management. It also abstracts away the protocol: the code can change without affecting the client/server behavior.

#### 4.2.3. Auxiliary Module

The Auxiliary module includes the set of functions required to control the execution of the TR program. It includes functions to maintain the execution of a function, to obtain the priority of the conditions and to compare values from the BS. Therefore, these functions can be classified into three groups: functions that control the execution of actions, functions that control priority of the rules and other functions. The durative actions are implemented with two discrete actions that represent the start and end of the action. Since the TeleoR conditions are continually evaluated, an implementation is needed to control the execution of the actions. In this way, the re-execution of actions already initiated is avoided (e.g., when the condition of the action is still true), as defined in the TR paradigm. The API functions, working as an interface, are listed in Table 13. 

Durative actions are executed through the execute_while/1 function during a certain amount of time or while a condition is true. Discrete actions are executed once through the execute/1 function. The function remember/2 keeps a belief in the BS during a certain amount of time specified as a parameter. The function compare_value/4 compares two values, which belong to two separate lists and returns true if the condition is true. The function add_timeout/3 is used to trigger a timeout in such a way that a belief is added to the BS if the action has not been executed before the deadline. The function while_condition/1 is used to maintain the execution of a rule for a given time, despite of the evaluation of its condition. The implementation of the whole module can be seen in the GitHub repository. 

Table 14 summarizes the correspondences between TR programs and Erlang (for more details about the implementation decision readers can access) [23].

#### 4.2.4. An MDE Based TR Editor 

**Figure 5:**
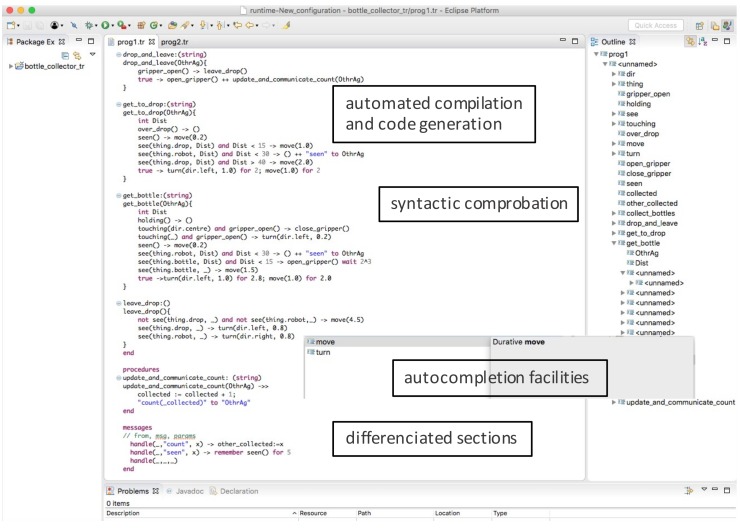
TR program editor implemented using Eclipse, Xtext and Xtend.

Figure 5 shows a screenshot of the tool developed using Eclipse Xtext and Xtend [24] facilities for creating a domain specific language for a subset of the TeleoR syntax. The user can edit a TR program with syntax validation and afterwards can generate automatically the Erlang code automatically for the Raspberry Pi devices. A configuration file is used to set the correspondences between the input/outputs of the IoT device with the percepts/actions of the TR agent. 

Although not executed at this time, the tool will allow the type of device to be selected for the deployment. The following section describes how the implementation is done on a Raspberry Pi device.

## 5. Validation of the Approach: Deployment on a Raspberry Pi

To test our TR node and the functionalities of the Erlang interpreter we made a deployment to emulate the bottle collecting robot. First, the details of the hardware and software implementation are given, followed by an analysis of the performance and consumption of resources. 

### 5.1. Hw/Sw Implementation

**Figure 6:**
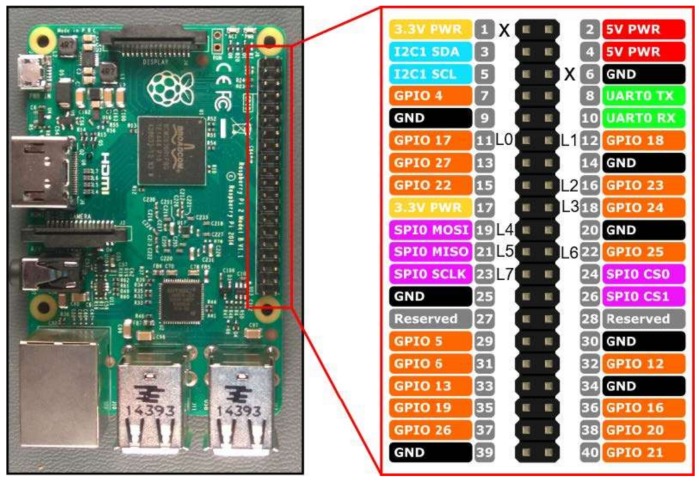
Raspberry Pi used pins scheme.

In order to simulate the robot and its environment, a hardware platform compatible with the infrastructure described in Figure 2 is needed. A number of features must be assessed when selecting a hardware platform for a node [25], including: processor speed, networking capabilities, power consumption, number of GPIO, size and price. Our implementation was tested on a Raspberry Pi 2 Model B [26], although other platforms with similar features could be used. Raspberry Pi has three main advantages: good capabilities, the possibility of using a Linux-based Operative System and the existence of an Erlang Virtual Machine. As its main disadvantage is its relatively high price, in order to achieve a cost-effective design, it is recommended to move to a cheaper and more constrained customized platform. The emulation of the agent’s environment is accomplished by the use of LEDs and switches and the software layer consists of the Raspbian operative system and its libraries, the Erlang interpreter described here and the Erlang code using the previous transformation procedure. The Raspberry Pi pins used are the ones shown in Figure 6 and summarized in Table 15. Four LEDs indicate the current state of the robot (executing or not executing the move and turn, gripper status, and bottle collected), which can be changed by four switches (to indicate touching a bottle, seeing the drop, seeing a bottle, and being over the drop). 

**Figure 7:**
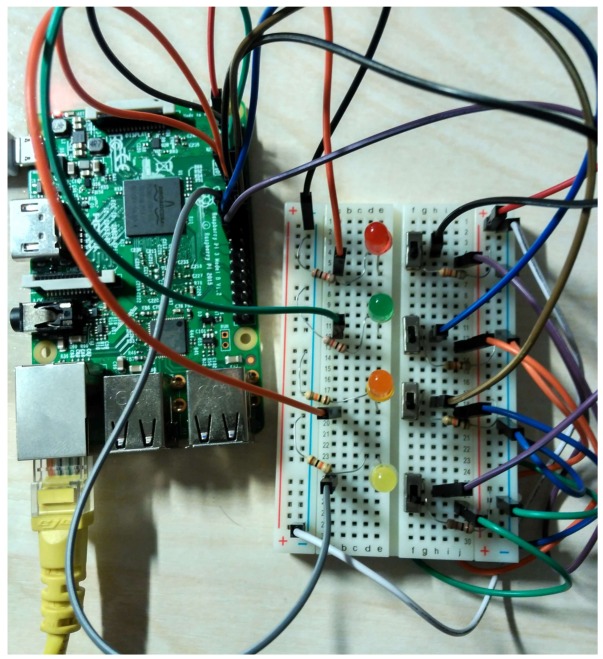
Final circuit for the simulation of a bottle collecting robot using a TR node.

Figure 7 shows the final circuit of the example. From top to bottom, the colors of the LEDs are: red, green, orange and yellow. The switches, from top to bottom, represent the touching, see drop, see bottle, and over drop states.

**Figure 8:**
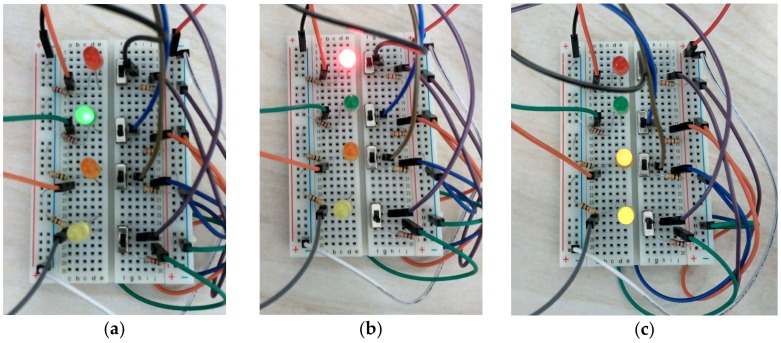
Different states of the robot as a result of the emulation: (**a**) alternating between turning and moving; (**b**) moving forward; (**c**) over the drop and openning the gripper

Figure 8 shows the emulation accomplished. The three states are: (a) there are no percepts yet (BS=[]), the robot is alternating between the turn and move actions looking for a bottle; the color green is just at the instant of turning; (b) the user pushes the switch C (BS=[see_bottle]) and then the robot moves forward (red LED) until the user pushes the switch A, indicating that the robot is touching the bottle (BS=[see_bottle, touching]); the robot then opens and closes the gripper to obtain the bottle (these actions and the percept holding have not been included in the hardware emulation); (c) once the user pushes the switch D the robot is over the drop and then opens the gripper, updating the variable _collected (orange and yellow LEDs).

### 5.2. Comparative Execution Results

We performed various experiments to compare the computational performance of the Erlang implementation with an implementation of the same example using the Python programming language and adopting the easiest sequential solution. Both implementations are included in the referenced GitHub repository. With the aim of analyzing the different performances of the Erlang and Python implementations, a test was carried out with different states and events. During the test, the CPU load and the memory usage of the Raspberry Pi were stored by the *RPi-Monitor* (http://rpi-experiences.blogspot.com.es/), which provides an interactive web interface to display the status of the Raspberry Pi and graphs of several parameters such as CPU load, memory usage, temperature and volume of data exchanged on the network.

**Figure 9:**
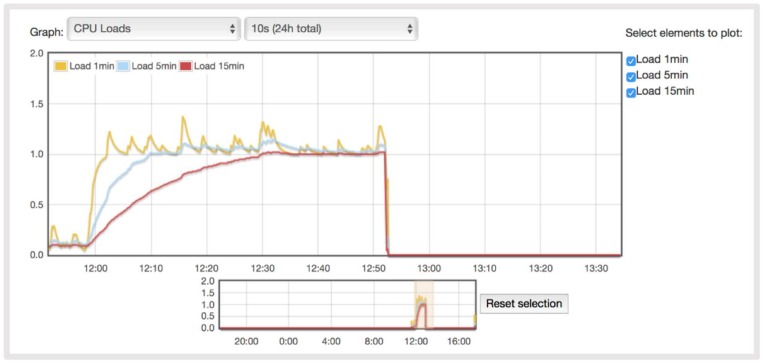
Raspberry Pi CPU load with the Erlang implementation.

**Figure 10:**
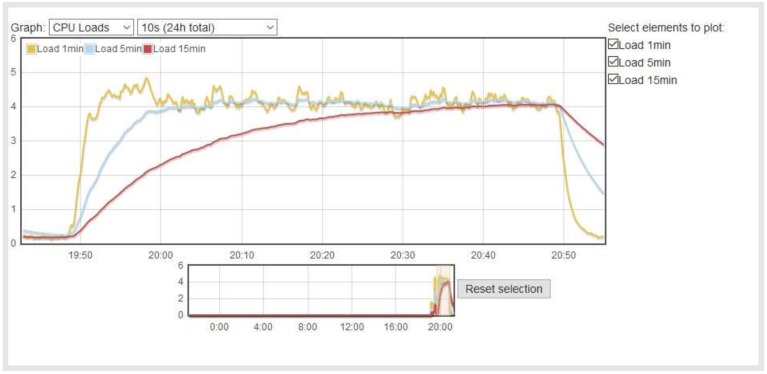
Raspberry Pi CPU load with the Python implementation.

Figure 9 and Figure 10 show the CPU load during the test. Each process using or waiting for CPU increments the load number by 1. The three load average curves refer to the last one, five, and fifteen minutes of system operation. They all decay exponentially, but at different speeds (they decay exponentially by *e* after 1, 5, and 15 min respectively). As the Raspberry Pi is a single-CPU system, the load average is a measure of system utilization during the respective time period. The Erlang implementation uses the CPU more than the Python, due to the concurrency of Erlang. In this implementation, the system is overloaded by 300 percent on average so that 3 processes had to wait for a turn. The overload is similar for the 5- and 15-min load averages. In the Python implementation, the system is overloaded by 25 percent on average. This difference is due to the Python implementation being sequential, while Erlang has several concurrent processes, which enables faster reactions in dynamic environments but also implies a higher CPU load. 

**Figure 11:**
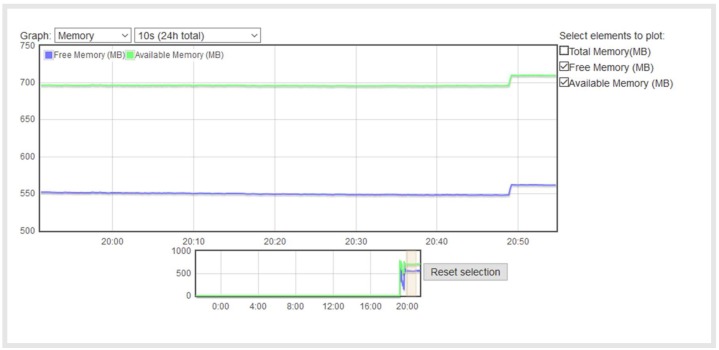
Raspberry Pi RAM memory with the Erlang implementation.

**Figure 12:**
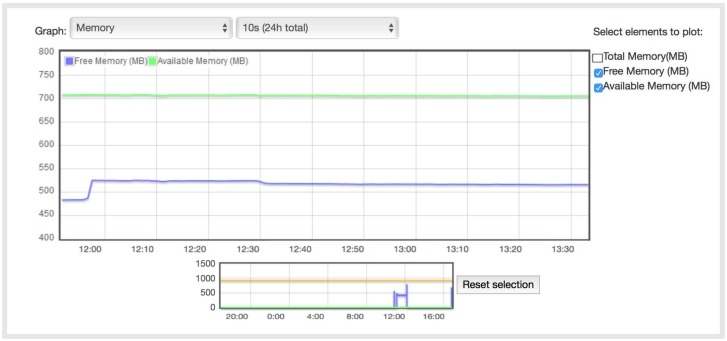
Raspberry RAM memory with the Python implementation.

Figure 11 and Figure 12 show the available Raspberry Pi RAM memory, where the total memory is 1 GB. It is shown that the memory usage in both cases is almost the same: 550 MB of free memory and 700 MB of available memory (the free memory is memory that is not being used, but it is actually harder to use because it has to be transitioned from free memory to memory in use, while the available memory can easily be switched to another use).

Table 16 shows the results of the Raspberry Pi’s power consumption for the robot example for both implementations. The HAMEG HM7042-5 Triple Power Supply was utilized. The measures consider the consumption of HDMI connector and LEDs (~25 mA and ~5 mA per LED, respectively). Considering that the above differences are insignificant, developers should choose the novel approach, due to the facilities provided to implement and evolve the system.

## 6. Discussion

The use of the TR approach for IoT nodes seems a feasible approach due to their common characteristics (especially when dealing with unreliable and non-distributed networks) and helps to simplify the specification of these complex systems. The TR nodes constantly sense the environment, detect changes and react accordingly while pursuing their final goal. If something goes wrong, they do not fail but go back a few steps to stubbornly evolve again towards their final goal. In a TR network this means that even if some nodes fail or disconnect, when properly configured, the network will continue to move forward to its final goal. 

**Figure 13:**
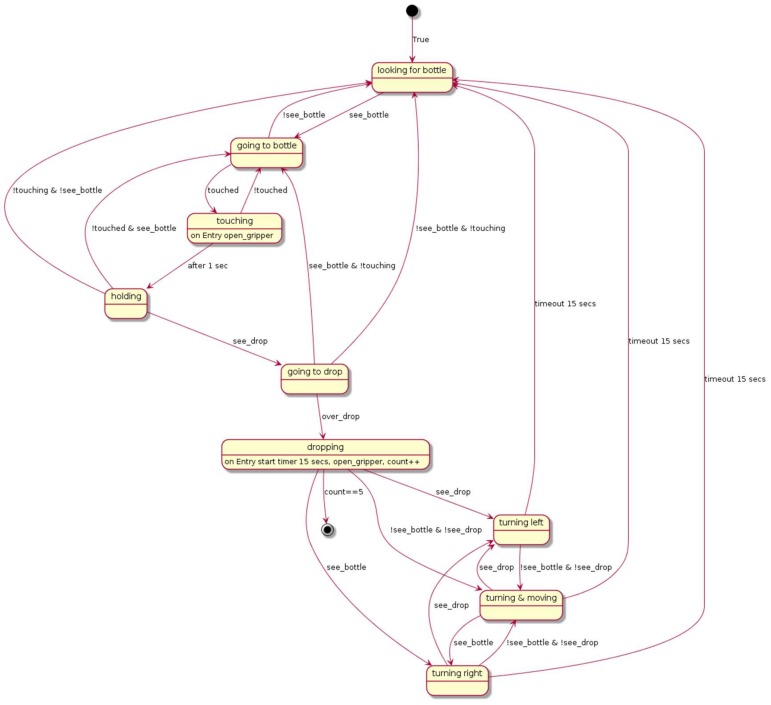
Statechart representing the behaviour implemented for the robot example.

In terms of power consumption, the Erlang implementation is similar to the Python one. However, in comparison with ordinary programs, the Erlang version demands more memory and CPU resources. Nevertheless, we believe that the advantages of the approach highly outweigh its disadvantages. Figure 13 shows the difficulties when implementing the example using only the basic programming facilities of the device with the statechart diagram equivalent to the code implemented in Python for the bottle collecting robot example. As can be seen, any modification of the implementation is error prone and difficult. By way of example, it would be quite easy to modify the TR program by switching the priority of two rules in the leave_drop() subgoal from:

leave_drop(){
    not see(drop) & not see(bottle) ~> move(1.0) 
    see(drop) ~> turn(left, 0.8)
    see(bottle)  ~> turn(right, 0.8)
    }
to
leave_drop(){
    not see(drop) & not see(bottle) ~> move(1.0)
    see(bottle)  ~> turn(right, 0.8) 
    see(drop) ~> turn(left, 0.8)
    }
      

This direct change in the TR program is not easy to do in the statechart, as many transitions will be affected (in the upper-left part of the statechart). The changes would be more difficult in terms of the Python programming language. 

At the same time, our background in the TR paradigm of having a visual method to specify reactive systems complements the current contribution by giving a full framework for developing IoT systems and integrating the different roles of stakeholders (for a comprehensive view of how to specify TR-based systems using goal-oriented modeling techniques see [27]).

Regarding the validity of the considered case study, some issues could have threatened the validity of the experiment. Wohlin presented in [28] some recommendations for analyzing such threats from several points of view:

- Conclusion validity: In the experiment to compare both implementations, the TR program demands longer computation times and higher memory but the same power. The test was not performed to demonstrate that Erlang was better than Python programming language, but to identify really which approach provided better support for the specification of IoT systems. A threat to the validity of the results could be the programmer’s knowledge of the languages used (TeleoR vs. general-purpose programming languages). The experiment compares two implementations carried out by an IoT developer who knows both languages. The experiment could be repeated with users who do not know any of the two languages and even make comparisons between implementations with a third language.
- Internal validity: The case study was complex enough to test the necessary mechanisms when implementing IoT applications (many sensors and actuators, message exchanging with other agents, etc.). A threat to the validity of the results could be the fact of not considering all types of TR nodes. The use of all levels of the hierarchy should be incorporated in future experiments. The feasibility from a technological point of view has been demonstrated in Section 4.2.1.
- Construct validity: As construct validity is mainly related to the method used to evaluate the outcomes of the task of the experiment, some threats were avoided by using RPi-Monitor that enjoys reputation for Raspberry Pi resources usages.

## 7. Conclusions

The TR approach has attracted a great deal of interest from researchers in recent years. Stakeholders do not have to deal with complex algorithms and programming languages; they only need to define the behavior as a set of rule-based goals. This allows non-professional developers to be involved in the specification of their own systems and thus will help to advance the IoT landscape. In other words, the TR program is the whole specification of the IoT system and does not need complementary views of the system to separate concerns into structural and behavioral issues. Apart from the simpler IoT system specification, TR nodes have other advantages. When many of them work together and adopt different hierarchical positions, highly robust, fault tolerant and hence, reliable networks of IoT devices can be deployed.

Future lines of research will be oriented towards deploying the TR nodes in other real life scenarios to assess their performance and specify more complex IoT systems in different domains using heterogeneous devices. Furthermore, we will make an experiment using a questionnaire with experienced developers to analyze the understandability of TR specifications using the approach. The specification of the systems and the questionnaires used could be reviewed by several external experts to avoid a possible source of bias. Besides, we are also developing a graphical user interface to make programming and configuring the TR nodes even simpler. Users will be able to specify the behavior of their nodes from a tablet by simply choosing the sensors, actuators and services from a customizable list by clicking and dragging the TR rules that determine the behavior of the node and its relationship with other nodes. This will also facilitate the reuse of specifications by means of catalogues. 

## Figures and Tables

**Table 1:** Structure of a generic TR program.

Condition		Action
*K*_1_	→	*a*_1_
*K*_2_	→	*a*_2_
	…	
*K_i_*	→	*a_i_*
	…	
*K_m_*	→	*a_m_*

**Table 2:** TeleoR specification of a bottle collecting robot.

%% ================================================================================== %% %% Bottle Collecting Robot %% %% ================================================================================== %% **enums** dir ::= left | right %% dir and thing are enums thing ::= bottle | drop **end** **percepts** %% directly linked to sensors gripper_open: (), holding: (), see: (thing), touching: (), over_drop: () **end** **beliefs** %% other beliefs. None in the current example **end** **durative** %% durative actions (actuators) move : (double), turn : (dir) **end** **discrete** %% ballistic actions (actuators) open_gripper: (), close_gripper: () **end** **vars** int _collected := 0 %% an internal variable to keep the account of bottles int _other_collected := 0 %% account of bottles of the other robot **end** **goals** collect_bottles:(string) %% main goal of the TR program where the execution starts collect_bottles(OtherAg){ _collected + _other_collected >= 10 ~> () %% do nothing holding & over_drop ~> drop_and_leave(OtherAg) holding ~> get_to_drop() true ~> get_bottle() } drop_and_leave:(string) drop_and_leave(OtherAg){ gripper_open ~> leave_drop() %% update _collected variable and send msg to the other robot true ~> open_gripper() ++ _collected := _collected + 1; count (_collected) to OtherAg } leave_drop:() leave_drop(){ not see(drop) & not see(bottle) ~> move(1.0) see(drop) ~> turn(left, 0.8) see(bottle) ~> turn(right, 0.8) } get_to_drop:() get_to_drop(){ over_drop ~> () see(drop) ~> move(1.5) %% turn left for 2s, then move for 2s and repeat true ~> turn(left,1.0) for 2; move(1.0) for 2 } get_bottle:() get_bottle(){ holding ~> () touching & gripper_open ~> close_gripper() touching ~> open_gripper() see(bottle) ~> move(1.5) true ~> turn(left,1.0) for 2.8; move(1.0) for 2 } **end**
**messages** %% from, msg, params handle(_,”count”, val) -> _other_collected := val %% update the variable with the param handle(_,_,_) %% do nothing for other messages **end**

**Table 3:** From TeleoR goal (left) to Erlang function (right).

task1:() ~> task1(){ Condition1 ~> Action1 . . . ConditionN-1 ~> ActionN-1 true ~> ActionN }	task1()-> case {Condition1, ..., ConditionN-1} of {true,_,...,_} -> Action1; . . . {_,_,...,true} -> ActionN-1; {_,_,...,_} -> ActionN end.

**Table 4:** From TeleoR percept/belief (left) to Erlang equivalent (right): general scheme.

task1:() ~> task1(){ Percept1 ~> Action1 . . . BeliefN-1 ~> ActionN-1 true ~> ActionN }	task1()-> case {bs:is_belief(Percept1),..., bs:is_belief(BeliefN-1} of {true,_,...,_}-> Action1; . . . {_,_,...,true}-> ActionN-1; {_,_,...,_} -> ActionN end.

**Table 5:** From TeleoR percept/belief (up) to Erlang equivalent (down): bottle collecting robot.

leave_drop:() ~> leave_drop (){ not **see(drop)** & not **see(bottle)** ~> move(1.0) %% {false,false} **see(drop)** ~> turn(left, 0.8) %% {_,true} **see(bottle)** ~> turn(right, 0.8) %% {true,_} }
leave_drop() -> case {bs:is_belief(**see,bottle**),bs:is_belief(**see,drop**)} of {false,false} -> %% execute_while because it is a durative action. %% {leave_drop,not_see} to register the goal and rule initiating the action %% move is the action and {1.0} is its parameter %% true is indicating that there is no time limit for the action execution %% collect_bottles() is a parameter to know the main goal auxilary:execute_while([{leave_drop,not_see},move,{1.0},true]), collect_bottles(); {_,true} -> auxilary:execute_while([{leave_drop,see_drop},turn,{left,0.8},true]), collect_bottles(); {true,_} -> auxilary:execute_while([{leave_drop,see_bottle},turn,{right,0.8},true]), collect_bottles() end.

**Table 6:** From TeleoR condition on variables (left) to Erlang equivalent (right).

task1:()~> task1(){ var1 + var2 < A ~> Action1 percept1(C) & C < F ~> Action2 true ~> Action3 }	task1()-> case { (bs:get_belief(var1)+bs:get_belief(var2))<A, auxilary:compare_value(C,bs:get_belief(percept1),minor,F)} of {true,_} -> Action1; {_,true} -> Action2; {_,_} -> Action 3 end.

**Table 7:** From TeleoR condition on variables (up) to Erlang equivalent (down).

collect_bottles(){ _collected + _other_collected >= 10 ~> () %% do nothing holding & over_drop ~> drop_and_leave holding ~> get_to_drop true ~> get_bottle }
collect_bottles()-> case {bs:get_belief(_collected)+ bs:get_belief(_other_collected)>=10, bs:is_belief(holding) & bs:is_belief(over_drop), bs:is_belief(holding)} of {true,_,_}-> nil; {_,true,_}-> drop_and_leave(bs:get_belief(otherAg)); {_,_,true}-> get_to_drop(); {_,_,_} -> get_bottle() end.

**Table 8:** TeleoR variable update (left). Erlang variable update (right).

variable1 := variable1 + 1	bs:update_belief({variable1, variable1 + 1})

**Table 9:** TeleoR variable update (up). Erlang variable update (down).

drop_and_leave:(string) ~> drop_and_leave(OtherAg){ gripper_open ~> leave_drop %% update _collected variable true ~> open_gripper() ++ **_collected := _collected + 1**; count (_collected) to OtherAg }
drop_and_leave(OtherAg) -> case bs:is_belief(gripper_open) of true -> leave_drop(); _ -> auxilary:execute([{drop_and_leave,true},open_gripper,[]]), ** bs:update_belief({collected,bs:get_belief(_collected)+1}),** OtherAg ! {count,bs:get_belief(_collected)} collect_bottles() end.

**Table 10:** TeleoR managing messages (up) and Erlang equivalent (b).

... ++ **count**(_collected) **to OtherAg** ... messages %% from, msg, params ** handle**(_,”count”, val) -> _other_collected := val %% update the variable with the param handle(_,_,_) %% do nothing for other messages end
drop_and_leave()-> ... {_} -> executor:execute([{drop_and_leave,true},open_gripper,[]]), bs:update_belief({_collected,bs:get_belief(_collected)+1}), ** OtherAg ! {count**,**bs:get_belief(_collected)}**,... ... handle_message()-> receive {_,**count**,**Num**}-> bs:update_belief({**_other_collected,Num**}), handle_message(); %% recursive call: always waiting for incoming messages {_,_,_}-> handle_message() end.

**Table 11:** Functions managing the BS.

**-module(bs).** %% This tell Erlang that we’ll be using gen_server module for the behavior. **-behaviour(gen_server).** %% ==================== %% API Function Exports %% The number is the arity of the function %% ==================== -export([start_link/0, add_belief/1, update_belief/1, remove_belief/1, remove_one_belief/1, get_belief/1, get_bs/0, is_belief/1, is_belief/2, stop/0]). %% ==================================== %% *gen_server* Function Exports, %% are wrappers for calls to the server %% ==================================== -export([init/1, %% called when a connection is made to the server handle_call/3, %% called in response to gen_server:call handle_cast/2, handle_info/2, terminate/2, code_change/3]).

**Table 12:** API functions definitions for the BeliefStore module.

%% ======================== %% API Function Definitions %% ======================== start_link() -> gen_server:start_link({local, ?MODULE}, ?MODULE, [], []). add_belief(Belief)-> gen_server:cast(?MODULE,{add, Belief}). update_belief(Belief)-> gen_server:cast(?MODULE, {update, Belief}). remove_belief(Belief)-> gen_server:cast(?MODULE, {remove, Belief}). remove_one_belief(Belief)-> gen_server:cast(?MODULE, {remove_one, Belief}). get_belief(Belief)-> gen_server:call(?MODULE,{get, Belief}). is_belief(Key)-> gen_server:call(?MODULE,{is_belief, Key}). is_belief(Key1, Key2)-> gen_server:call(?MODULE,{is_belief, Key1, Key2}). get_bs()-> gen_server:call(?MODULE,{get_bs}). stop()-> gen_server:cast(?MODULE, stop).

**Table 13:** API functions that work as an interface.

**-module(auxilary).** %% ============= %% API functions %% ============= -export([execute_while/1, execute_while/5, execute/1, add_timeout/3, remember/2, compare_value/4, while_condition/1]).

**Table 14:** Main correspondences between TR programs and the Erlang implementation.

Concept in TR Program	Artifact in Erlang
goal	function declaration with *case* expression
rule	function clause
rule condition	guard sequence
rule action	function call
variable, percept, belief	variable accessible through the BS
arithmetic expression	arithmetic expression on function calls accessing the BS
assignment statement in rule	function call updating the BS
message to another agent	message to an Erlang process
message handle	clause of the *handle_message* function
remember/forget operation	ad hoc implementations of Erlang functions for accessing the BS
*while* condition in rule	ad hoc implementations of Erlang functions for extending the rule conditions
timed sequence of rule durative actions	sequence of function calls triggering alarms for finishing the action execution
enums	atoms

**Table 15:** Pins, attached devices and associated actions/states for the Raspberry Pi circuit.

Pin	I/O Attached	Action	States
L0	Red LED	move	
L1	Green LED	turn	
L2	Orange LED		gripper is open
L4	Yellow LED		bottle is collected
L3	Switch A		touching
L5	Switch B		see drop
L6	Switch C		see bottle
L7	Switch D		over drop

**Table 16:** Power consumption for both implementations.

Version	Pi State	Power Consumption (Input 5 V)
Python	powering on	100–440 mA (0.5–2.2 W)
	idle	320–330 mA (1.6–1.65 W)
	running the robot example	340–370 mA (1.7–1.85 W)
Erlang interpreter	powering on	107–400 mA (0.535–2 W)
	idle	310–330 mA (1.55–1.65 W)
	running the robot example	414–450 mA (2.07–2.25 W)

## References

[1] Gubbi J., Buyya R., Marusic S., Palaniswami M. (2013). Internet of Things (IoT): A vision, architectural elements, and future directions. Future Gener. Comput. Syst..

[2] Mulani T., Pingle S. (2016). Internet of Things. Int. Res. J. Multidiscip. Stud. SPPP’s.

[3] Atzori L., Iera A., Morabito G. (2010). The Internet of Things: A survey. Comput. Netw..

[4] Chen C., Helal S., de Deugd S., Smith A., Chang C.K. Toward a collaboration model for smart spaces. Proceedings of the 2012 3rd International Workshop on Software Engineering for Sensor Network Applications, SESENA 2012.

[5] Patel P., Cassou D. (2015). Enabling high-level application development for the Internet of Things. J. Syst. Softw..

[6] Nilsson N. (1994). Teleo-Reactive programs for agent control. J. Artif. Intell. Res..

[7] Nilsson N. TR Programs. http://teleoreactiveprograms.net.

[8] Morales J.L., Sánchez P., Alonso D. (2014). A systematic literature review of the teleo-reactive paradigm. Artif. Intell. Rev..

[9] Clark K.L., Robinson P.J. Robotic agent programming in TeleoR. Proceedings of the 2015 IEEE International Conference on Robotics and Automation (ICRA).

[10] Sánchez P., Álvarez B., Morales J.M., Alonso D., Iborra A. (2016). An approach to modeling and developing teleo-reactive systems considering timing constraints. J. Syst. Softw..

[11] Qulog/TeleoR Home Page. http://staff.itee.uq.edu.au/pjr/HomePages/QulogHome.html.

[12] The Object Management Group Website. http://www.omg.org/spec/UML.

[13] Kent S., Butler M., Petre L., Sere K. (2002). Model Driven Engineering. Integrated Formal Methods. IFM 2002.

[14] Cassou D., Bruneau J., Consel C., Balland E. (2012). Towards a Tool-based Development Methodology for Pervasive Computing Applications. IEEE TSE Trans. Softw. Eng. IEEE Comput. Soc..

[15] Serral E., Valderas P., Pelechano V. (2010). Towards the model driven development of context-aware pervasive systems. Pervasive Mob. Comput..

[16] Cubo J., Brogi A., Pimentel E. Behaviour-aware compositions of things. Proceedings of the IEEE International Conference on Green Computing and Communications.

[17] Cubo J., Brogi A., Pimentel E. (2014). A cloud-based Internet of Things platform for ambient assisted living. Sensors.

[18] Fernández D., Sánchez P., Álvarez B., Riquelme J.A., Iborra A. (2017). TRIoT: A Proposal for Deploying Teleo-Reactive Nodes for IoT Systems. Advances in Cyber-Physical Multi-Agent Systems.

[19] Erlang Official Website. https://www.erlang.org/.

[20] Logan M., Merritt E., Carlsson R. (2010). Erlang and OTP in Action.

[21] Dongol B., Hayes I.J., Robinson P.J. (2014). Reasoning about goal-directed real-time teleo-reactive programs. Form. Asp. Comput..

[22] Erlang/OTP Documentation. http://erlang.org/doc/man/gen_server.html.

[23] Antolinos E. (2017). Erlang Implementation of a Virtual Machine for Executing Teleo-Reactive Programmes in the Internet of Things. Final Degree Thesis.

[24] Eclipse Xtext Documentation. https://www.eclipse.org/Xtext/.

[25] McEwen A., Cassimally H. (2014). Designing the Internet of Things.

[26] Raspberry 2 Model B Features. https://www.raspberrypi.org/products/raspberry-pi-2-model-b/.

[27] Morales J.M., Navarro E., Sánchez P., Alonso D. (2016). A family of experiments to evaluate the understandability of TRiStar and i* for modeling teleo-reactive systems. J. Syst. Softw..

[28] Wohlin C., Runeson P., Höst M., Ohlsson M.C., Regnell B., Wesslén A. (2000). Experimentation in Software Engineering: An Introduction.

